# Children With Short Stature Display Reduced *ACE2* Expression in Peripheral Blood Mononuclear Cells

**DOI:** 10.3389/fendo.2022.912064

**Published:** 2022-07-15

**Authors:** Federica Tonon, Gianluca Tornese, Fabiola Giudici, Francesca Nicolardi, Barbara Toffoli, Egidio Barbi, Bruno Fabris, Stella Bernardi

**Affiliations:** ^1^ Department of Medical Surgical and Health Sciences, Ospedale di Cattinara, University of Trieste, Trieste, Italy; ^2^ Institute for Maternal and Child Health IRCCS ‘Burlo Garofolo’, Trieste, Italy; ^3^ Department of Cardiac, Thoracic, Vascular Sciences and Public Health, University of Padova, Padova, Italy; ^4^ Operative Unit of Medicina Clinica, Ospedale di Cattinara, Azienda Sanitaria Universitaria Giuliano Isontina (ASUGI), Trieste, Italy

**Keywords:** ACE2, growth hormone deficiency, short stature, growth retardation, Renin-Angiotensin System, angiotensin, ANP

## Abstract

**Background:**

The cause of short stature remains often unknown. The renin-angiotensin system contributes to growth regulation. Several groups reported that angiotensin-converting enzyme 2 (*ACE2*)-knockout mice weighed less than controls. Our case-control study aimed to investigate if children with short stature had reduced *ACE2* expression as compared to controls, and its significance.

**Materials and Methods:**

children aged between 2 and 14 years were consecutively recruited in a University Hospital pediatric tertiary care center. Cases were children with short stature defined as height SD ≤ −2 diagnosed with growth hormone deficiency (GHD) or idiopathic short stature (ISS), before any treatment. Exclusion criteria were: acute diseases, kidney disease, endocrine or autoimmune disorders, precocious puberty, genetic syndromes, SGA history. *ACE* and *ACE2* expression were measured in peripheral blood mononuclear cells, angiotensins were measured by ELISA.

**Results:**

Children with short stature displayed significantly lower *ACE2* expression, being 0.40 fold induction (0.01-2.27) as compared to controls, and higher *ACE/ACE2*, with no differences between GHD and ISS. *ACE2* expression was significantly and inversely associated with the risk of short stature, OR 0.26 (0.07-0.82), and it had a moderate accuracy to predict it, with an AUC of 0.73 (0.61-0.84). The cutoff of 0.45 fold induction of *ACE2* expression was the value best predicting short stature, identifying correctly 70% of the children.

**Conclusions:**

Our study confirms the association between the reduction of *ACE2* expression and growth retardation. Further studies are needed to determine its diagnostic implications.

## Introduction

Short stature is one of the most common reasons parents seek consultation with a growth specialist (1). Despite standard clinical and laboratory evaluation, a pathological cause is usually not found in up to 50-90% of cases, and children are eventually diagnosed as having constitutional delay of growth, familial short stature, or idiopathic short stature (2). Growth regulation is important not only per se but also because it seems associated with adult disease. In particular, fetal, infant, and childhood growth are predictors of coronary heart disease, diabetes, and hypertension in adult men and women (3). It is well known that embryogenesis, fetal development, and post-natal growth are controlled by the coordinated action of different hormonal regulators. In addition to traditional growth hormones (GHRH/GH/IGF-1 axis), other peptide hormones, such as angiotensins, have been implicated in growth regulation (4).

The renin angiotensin system (RAS) is a pivotal regulator of vascular homeostasis. It is composed of different enzymes and peptides whose main function is the dynamic control of vascular function, blood pressure and fluid balance (5). Many of these components have opposing functions, such as angiotensin converting enzyme (ACE) that forms the vasoconstrictor Angiotensin II (AngII) and ACE2 that cleaves AngII, producing the vasodilator Angiotensin 1-7 (Ang1-7). In addition to the regulation of vascular function, AngII promotes inflammation, fibrosis and apoptosis, while Ang1-7 is associated with the opposite beneficial effects (5). Overall, RAS final effects depend on the activity of both ACE and ACE2, which determines the amount of circulating and tissue AngII and Ang1-7 (6). Interestingly, it has been argued that ACE2 may be even more important than ACE in some settings, such as the regulation of local levels of AngII and Ang1-7. For instance, in *ACE*-knockout mice, tissue AngII is not significantly modified because it is generated by non-ACE pathways (7), while in *ACE2*-knockout mice tissue AngII increases significantly, due to the lack of alternative pathways to ACE2 (8).

ACE2 was discovered in 2000 (9), and this was followed by the generation of *ACE2*-knockout mice to characterize its physiological functions. The first studies reported that these mice appeared healthy and fertile, apart from a marked defect in cardiac contractility that was observed by some Authors (8) and not by others (10), possibly due to a difference in *ACE2*-knockout genetic backgrounds. Further works were carried out in different laboratories to establish other functions of ACE2 (11–13). When we were studying the effects of ACE2 deficiency on glucose metabolism, we found that *ACE2*-knockout mice receiving a standard diet were smaller than wild-type mice, and this was not associated with differences in food intake, locomotor activity or heat production (13). Over time, several other groups have reported that *ACE2*-knockout mice weighed less than wild-type mice (14–19).

It remains to be clarified if there is an association between ACE2 deficiency and a defect of human growth. Based on this background, here we investigated if children with short stature displayed reduced expression of *ACE2* in peripheral blood mononuclear cells as compared to controls and its implications.

## Materials and Methods

### Study Design and Population

This is an observational case-control study, aiming to compare children with short stature (either idiopathic or due to growth hormone deficiency) to respective controls. Subjects were consecutively recruited between October 2019 and June 2021 among the children aged between 2 and 14 years referred to the Clinica Pediatrica of the Institute of Maternal and Child Health ‘Burlo Garofolo’. Cases were children with persistent short stature [height ≤ −2 standard deviations (SD)] after the second year of life, diagnosed with growth hormone deficiency or idiopathic short stature, before starting any treatment. GHD was diagnosed on the basis of failure to respond to 2 provocative tests of GH secretion (20). Controls were children with normal growth (height SD > −2 after the second year of life), mostly recruited among healthy children undergoing allergy testing. Exclusion criteria were history of any acute disease in the 3 weeks prior to enrollment, history of kidney disease, other endocrine or autoimmune disorders, precocious puberty, small for gestational age, as well as genetic syndromes. In particular, we excluded patients with dysmorphic features, major malformations, microcephaly, neurodevelopmental delay, intellectual disabilities, or skeletal dysplasia. Although the protocol was written before COVID-19 outbreak, after February 2020 we excluded also children with history of COVID-19 (including history of positive PCR test for SARS-CoV2 from nasal swab).

After providing the informed consent, children underwent a medical visit. History and anthropometric parameters were recorded. These included: birth weight, weight, height, body mass index (BMI), sitting height/height ratio (SH/H ratio), arm span and the ratio between upper and lower segment (U/L ratio), as well as systolic and diastolic blood pressure (SBP and DBP). Standard deviations (SD) of weight, height, and BMI were calculated with the Growth4 software and following the Italian growth charts reported by Cacciari et al. (21). The following laboratory parameters were also recorded: full blood count, erythrocyte sedimentation rate (ESR), glucose, creatinine and electrolytes, bicarbonate, alkaline phosphatase (ALP), albumin, TSH, free T4 (FT4), IGF-1, anti transglutaminase Ab and total IgA levels. Standard deviation of IGF-1 was calculated with the following formula: IGF-1 SD (Z-score)=[(log IGF-1 ng/L)-(log mean for age and sex)]/log mean SD (22). Then, all the children underwent a fasting blood sampling, after a day of rest, to collect 5 ml of whole venous blood and 5 ml of serum.

This study was conducted in accordance with the Declaration of Helsinki, and the protocol was approved by the Institutional Review Board and Ethics Committee (CEUR-2019-Sper-115).

### PBMC Isolation, Gene Expression Analysis, and ELISA

The gene expression of *ACE* and *ACE2* was measured in isolated peripheral blood mononuclear cells (PBMC). To isolate PBMC, blood samples were collected in EDTA-tubes and added to the same volume of Ficoll-PaqueTM Plus (Cytiva Sweden AB) and then centrifuged at 2400 rpm for 30 minutes at room temperature. The mononuclear cell layer that was obtained was used to extract RNA.

PBMC were homogenized with 500 μl of Trizol (Invitrogen) per 5*10^5^ cells. In order to isolate mRNA, 100 μl of chloroform/isoamyl alcohol were added to each tube and the samples were vortexed for 15 seconds and left at room temperature for 5 minutes. Then samples were centrifuged at 13000 rpm for 20 minutes at 4°C and the upper aqueous phase was carefully collected to new tubes. In order to precipitate RNA, 250 μl of isopropanol were added to each tube and the tubes were briefly vortexed and left at −20°C overnight. The day after, samples were centrifuged at 13000 rpm for 15 minutes at 4°C to pellet the RNA precipitate. The supernatant was then carefully discarded and RNA was washed with 1ml of 75% ethanol and then centrifuged for 15 minutes at 13000 rpm. The supernatant was entirely removed, the RNA was resuspended in 20 μl of RNAse-free water and incubated at 55°C for 5 minutes, before quantifying RNA. RNA was treated with DNAse to eliminate DNA contamination (#AM-1906, Ambion DNA-free product), and 1.2 μg of treated RNA were subsequently used to synthesize cDNA with Superscript First-Strand synthesis system for RT-PCR (Gibco BRL). The expression of *ACE*, *ACE2* and *AT1R* (AngII type1 receptor) was evaluated with the TaqMan Gene Expression Assay (Life Technologies). Fluorescence for each cycle was quantitatively analyzed by StepOnePlus real-time PCR system (Applied Biosystems). Gene expression was normalized to 18s (TaqMan), and reported as a ratio compared with the level of expression in controls, which were given an arbitrary value of 1.

Serum AngII (Elabscience, E-EL-H0326) and Ang1-7 (Elabscience, E-EL-H5518), were measured by ELISA, according to manufacturer’s instructions. Briefly, 50 μl of each standard or samples were added to the respective (AngII or Ang1-7) pre-coated plate and, immediately after, 50 μl of specific biotinylated detection antibody were added to each well. The plate was incubated at 37°C for 45 minutes. Fluid was aspirated and the plate washed for 3 times with the wash buffer before adding 100 μl of Avidin-Horseradish Peroxidases solution for 30 minutes at 37°C. Fluid was aspirated and plate washed for 3 times with wash buffer before 90 μl of substrate reagent for 15 minutes at 37°C, and then 50 μl of stop solution to end the reaction. Absorbance was taken at 450 nm.

### Statistical Analyses

Sample size was calculated with openepi.com. To detect a mean difference in *ACE2* expression of 2 cycles (SD = 2) with a two-sided significance level of 5% and power of 80% with equal allocation to two groups would require 20 patients in each group. Based on this estimate, we decided to double this number taking into account the presence of GHD and ISS patients.

All statistical analyses were carried out in R system for statistical computing (Version 4.0.2; R development Core Team, 2020). Statistical significance was set at p<0.05. Shapiro-Wilk test was applied to continuous variables to check for distribution normality. Quantitative variables were reported as median with range (min-max) or mean ± standard deviation, depending on distribution. Categorical variables were reported as absolute frequencies and/or percentages. Continuous variables were compared by Mann-Whitney test (and Kruskall-Wallis test) or student t-test (and ANOVA), depending on data distribution and number of groups. Two multivariate regression models were performed: the first linear regression was performed to evaluate factors influencing *ACE2* expression and the second logistic regression to investigate if *ACE2* expression could predict being a child with short stature. Receiver operating characteristic (ROC) curves were evaluated to investigate the level of discrimination of *ACE2* expression in predicting short stature. The area under the curve (AUC) was calculated, with higher values indicating better discriminatory ability. The optimal thresholds for *ACE2* to differentiate between cases and controls, were calculated using Youden’s index method. Sensitivity, specificity, and positive and negative predictive values with 95% confidence interval (CI) were then calculated (R packages: pROC and OpimalCutpoints).

## Results

### General Characteristics

A total of 39 cases and 35 controls were recruited, whose characteristics are reported in **Table 1**. Median age of cases was 11 (3-14) years and median age of controls was 7.5 (3-14) years, as most of them were recruited among healthy children undergoing allergy testing. There were no differences in the proportion of boys and girls. Groups differed in terms of standard deviations (SD) of weight, height, and BMI, which were significantly lower in the group of children with short stature. This is consistent with the report that idiopathic prepubertal short stature might be associated with low BMI (23, 24). Hemoglobin, ESR, glucose, creatinine, electrolytes, bicarbonate, alkaline phosphatase, albumin, TSH values were within reference ranges in both groups. Screening for coeliac disease was negative in both groups. Children with short stature displayed significantly lower IGF-1 (SD) levels, being -1.81 [-4.21, 0.5] as compared to 0.17 (-2.42, 2.24) in the control group.

**Table 1:** General characteristics of whole cohort.

	Controls (n = 35)	Cases (n = 39)	p-value
Age (year)	7.5 [3, 14]	11 [3, 14]	<0.001
M/F (%)	40/60	51/49	0.33
Height (SD)	0.06 [-1.92, 2.40]	-2.36 [-3.16, -2.00]	<0.001
Weight (SD)	0.12 [-2.65, 1.54]	-2.29 [-3.91, -0.9]	<0.001
BMI (SD)	0.2 [-2.48, 1.46]	-1,2 [-2.27, 0.16]	<0.001
SH/H ratio (SD)	0.24 [-2.25, 2.33]	0.42 [-1.55, 2.86]	0.21
U/L ratio	1.15 [0.93, 1.44]	1.11 [0.97, 1.34]	0.06
Arm span/H	0.97 [-0.06, 1.06]	0.98 [0.92, 1.04]	0.34
SBP (mmHg)	103 [84, 135]	95 [70, 129]	0.23
DBP (mmHg)	62 [51, 75]	60.5 [45, 84]	0.29
Hb (g/dL)	13 [11, 15]	13 [10, 15]	0.59
ESR (mm/h)	9 [2, 52]	12 [2, 83]	0.29
Glucose (mg/dL)	90 [73, 99]	85 [54, 104]	0.32
Creatinine (mg/dL)	0.40 [0.27, 0.56]	0.46 [0.26, 0.76]	0.30
Na^+^ (mEq/L)	139 [135, 142]	138 [130, 141]	0.61
K^+^ (mEq/L)	4.21 [3.91, 4.87]	4.33 [3.90, 5.01]	0.17
${\text{HCO}}_{3}^{-}$ (mEq/L)	24 [22, 28]	24 [20, 28]	0.88
Ca^2+^ (mg/dL)	9.89 [9.34, 11.03]	10.00 [9.53, 10.58]	0.21
Phosphate (mg/dL)	4.97 [3.38, 6.11]	4.57 [3.37, 5.48]	0.28
ALP (U/L)	235 [41, 458]	227 [35, 462]	0.31
Albumin (g/dL)	4.32 [3.87, 4.81]	4.41 [3.89, 4.87]	0.38
TSH (µU/mL)	1.81 [0.90, 4.46]	2.05 [0.46, 4.37]	0.75
FT4 (pg/mL)	9 [7, 13]	9 [6, 11]	0.32
IGF1 (µg/L)	154 [49, 436]	122 [48, 242]	0.02
IGF1 (SD)	0.17 [-2.42, 2.24]	-1.81 [-4.21, 0.5]	<0.001

Data are expressed as median (min-max). SD is for standard deviation.

### Renin-Angiotensin System Expression

**Figure 1:**
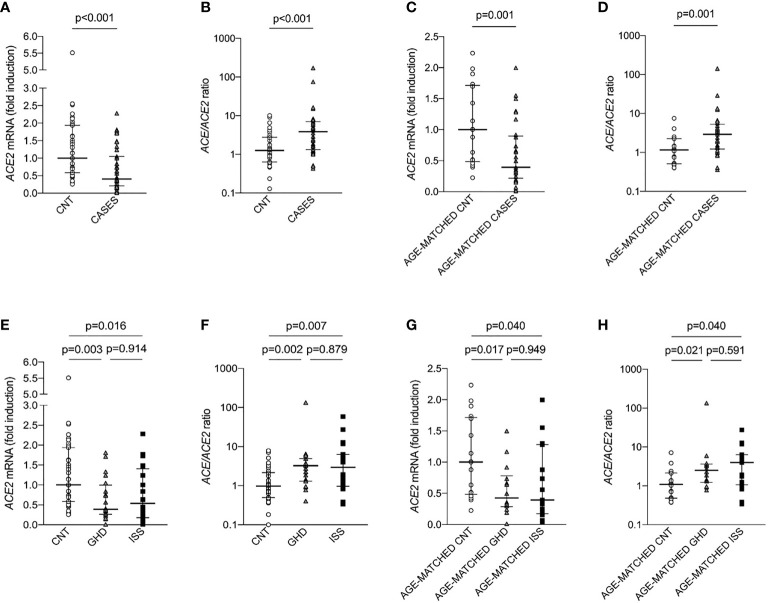
*ACE2* expression and *ACE*/*ACE2* ratio in children with short stature (CASES) and controls (CNT). Gene expression was measured as mRNA fold induction as compared to controls (CNT). *ACE* is for angiotensin converting enzyme, *ACE2* is for angiotensin converting enzyme 2, GHD is for growth hormone deficiency and ISS is for idiopathic short stature. **(A–D)** Mann-Whitney test, **(E–H)** Kruskall-Wallis test.

Also *ACE2* gene expression was significantly lower in the group of children with short stature, being 0.40 (0.01-2.27) fold increase in cases and 1.00 (0.25-5.49) in controls (p-value <0.001), **Figure 1A**. Consistent with this, *ACE*/*ACE2* ratio was significantly higher in the group of children with short stature, being 3.85 (0.43-172.07) in cases vs 1.2 (0.13-10.11) in controls (p-value <0.001), **Figure 1B**. There were no differences in terms of *ACE* and *AT1R* expression, as well as AngII and Ang1-7 circulating levels and their ratio between the two groups.

### Subgroup Analyses

To exclude that the difference in *ACE2* expression was confounded by the age of cases and controls, we identified a subgroup of 29 cases and a subgroup of 17 controls, who were matched by age (**Table 2**). Median age of cases was 11 (5-14) years and median age of controls was 9 (5-14) years. There were no differences in the proportion of boys and girls. Cases exhibited significantly lower weight (SD), height (SD), BMI (SD), IGF-1 levels (SD). In line with our previous results, children with short stature displayed significantly lower *ACE2* expression, being 0.39 (0.01-1.99) fold increase in cases as compared to 1 (0.22-2.23) in controls (p-value <0.01), **Figure 1C**. Consistent with this, *ACE*/*ACE2* ratio was significantly higher in the group of children with short stature, being 2.91 (0.36-142.21) in cases and 1.16 (0.40-7.57) in controls (p-value <0.01), **Figure 1D**. There were no differences in terms of *ACE* expression, AngII and Ang1-7 circulating levels as well as their ratio between the two groups.

**Table 2:** General characteristics of age-matched subgroups.

	Controls (n = 17)	Cases (n = 29)	p-value
**Age (year)**	9 [5, 14]	11 [5, 14]	0.55
**M/F (%)**	53/47	48/52	0.76
**Height (SD)**	0.04 [-1.92, 2.40]	-2.46 [-3.16, -2.00]	<0.001
**Weight (SD)**	-0.21 [-2.65, 1.54]	-2.24 [-3.77, -0.91]	<0.001
**BMI (SD)**	-0.20 [-2.48, 1.37]	-1.21 [-2.27, 0.12]	0.006
**IGF1 (µg/L)**	193 [90, 436]	127 [52, 242]	0.003
**IGF1 (SD)**	-0.11 [-2.42, 2.24]	-1.57 [-2.81, 0.50]	<0.001

Data are expressed as median (min-max). SD is for standard deviation.

### Idiopathic Short Stature and Growth Hormone Deficiency

Children with short stature were further divided into two subgroups: children with growth hormone deficiency (GHD, n=19), and children with idiopathic short stature (ISS, n=20). These two subgroups did not differ in terms of height, weight, and BMI. It has to be noted that not only GHD but also ISS is regarded as a disorder of the GH-IGF-1 axis, falling between GH deficiency and GH insensitivity in the so-called GH-IGF-1 axis continuum model (25), but differing from GHD for the response to GH stimulation test (20, 25). As compared to controls, *ACE2* expression was significantly reduced in both groups and there were no differences between children with GHD and children with ISS (p=0.914), **Figure 1E**. Also, *ACE/ACE2* ratio was significantly increased in both groups and there were no differences between the two groups (p=0.879), **Figure 1F**. These results were maintained also after matching the children for age (**Figures 1G, H**).

### Regression Analyses

To investigate the relationship between being a children with short stature and *ACE2*, as the response variable, we performed linear regression analyses taking into account the whole cohort (35 controls and 39 cases) as well as the subgroups matched by age (17 controls and 29 cases). Our data showed that *ACE2* expression was significantly and independently correlated to belonging to the short stature group as well as sex, while it was not correlated to age or BMI (**Table 3**). *ACE*/*ACE2* expression did not show any correlation with age, sex, and short stature. Then, to understand if *ACE2* expression was associated with the risk of short stature we performed a multivariate logistic regression using *ACE2* as a predictor variable and child status (control=0, case=1) as the response variable. The odds ratio (OR) for *ACE2* as predictor of belonging to the short stature group was 0.26, [95% confidence interval (CI) (0.07-0.82)], meaning that an increase of one unit in *ACE2* expression would be associated with a 74% decrease in the odds of being a child with short stature, regardless of age, sex, and BMI. This was maintained after matching for age. *ACE*/*ACE2* was associated with the risk of short stature only after matching for age.

**Table 3:** Regression models.

**A) LINEAR REGRESSION**				
**Dependent variable: *ACE2* expression**				
**WHOLE COHORT (35 cnt vs 39 cases)**				
Predictive variables	β-estimate	95%CI	Standard error	p-value
**Age**	0.02	[-0.04, 0.08]	0.03	0.56
**Sex [M]**	-0.80	[-1.15, -0.46]	0.13	<0.001
**Group [CNT]**	0.41	[-0.04, 0.87]	0.14	0.07
**BMI_SD**	0.11	[-0.11, 0.34]	0.11	0.31
**AGE-matched SUBGROUPS (17 cnt vs 29 cases)**				
Predictive variables	β-estimate	95%CI	Standard error	p-value
**Age**	-0.01	[-0.08, 0.05]	0.03	0.71
**Sex [M]**	-0.54	[-0.86, -0.22]	0.16	0.01
**Group [CNT]**	0.51	[0.14, 0.87]	0.18	0.01
**BMI_SD**	-0.03	[-0.22, 0.17]	0.09	0.77
**Dependent variable: *ACE*/*ACE2* expression**				
**WHOLE COHORT (35 cnt vs 39 cases)**				
Predictive variables	β-estimate	95%CI	Standard error	p-value
**Age**	-0.39	[-1.76, 0.96]	0.68	0.56
**Sex [M]**	3.07	[-4.93, 11.07]	4.01	0.45
**Group [CNT]**	-9.66	[-20.05, 0.73]	5.21	0.68
**BMI_SD**	1.58	[-3.57, 6.73]	2.58	0.54
**AGE-matched SUBGROUPS (17 cnt vs 29 cases)**				
Predictive variables	β-estimate	95%CI	Standard error	p-value
**Age**	0.22	[-2.48, 2.92]	1.34	0.87
**Sex [M]**	6.10	[-7.28, 19.50]	6.63	0.36
**Group [CNT]**	-9.48	[-25, 6.04]	7.68	0.22
**BMI_SD**	1.79	[-6.43, 10.01]	4.07	0.66
**B) LOGISTIC REGRESSION**				
**Dependent variable: CNT vs CASE**				
**WHOLE COHORT (35 cnt vs 39 cases)**				
Predictive variables	OR	95%CI		p-value
**Age**	1.25	[1.01, 1.59]		0.04
**Sex [M]**	0.83	[0.16, 3.97]		0.82
***ACE2* mRNA** **BMI_SD**	0.26 0.18	[0.07, 0.82] [0.07, 0.41]		0.03 <0.001
**AGE-matched SUBGROUPS (17 cnt vs 29 cases)**				
Predictive variables	OR	95%CI		p-value
**Age**	0.98	[0.68, 1.38]		0.92
**Sex [M]**	0.36	[0.03, 2.74]		0.36
***ACE2* mRNA**	0.10	[0.01, 0.55]		0.02
**BMI_SD**	0.21	[0.06, 0.54]		<0.01
**WHOLE COHORT (35 cnt vs 39 cases)**				
Predictive variables	OR	95%CI		p-value
**Age**	1.28	[1.02, 1.64]		0.04
**Sex [M]**	1.42	[0.29, 6.70]		0.65
***ACE*/*ACE2* mRNA**	1.27	[1.03, 1.92]		0.18
**BMI_SD**	0.19	[0.07, 0.42]		<0.001
**AGE-matched SUBGROUPS (17 cnt vs 29 cases)**				
Predictive variables	OR	95%CI		p-value
**Age**	1.01	[0.72, 1.40]		0.95
**Sex [M]**	0.35	[0.04, 2.54]		0.32
***ACE*/*ACE2* mRNA**	1.97	[1.15, 4.39]		0.04
**BMI_SD**	0.19	[0.04, 0.52]		<0.01

### ROC Analysis

**Figure 2:**
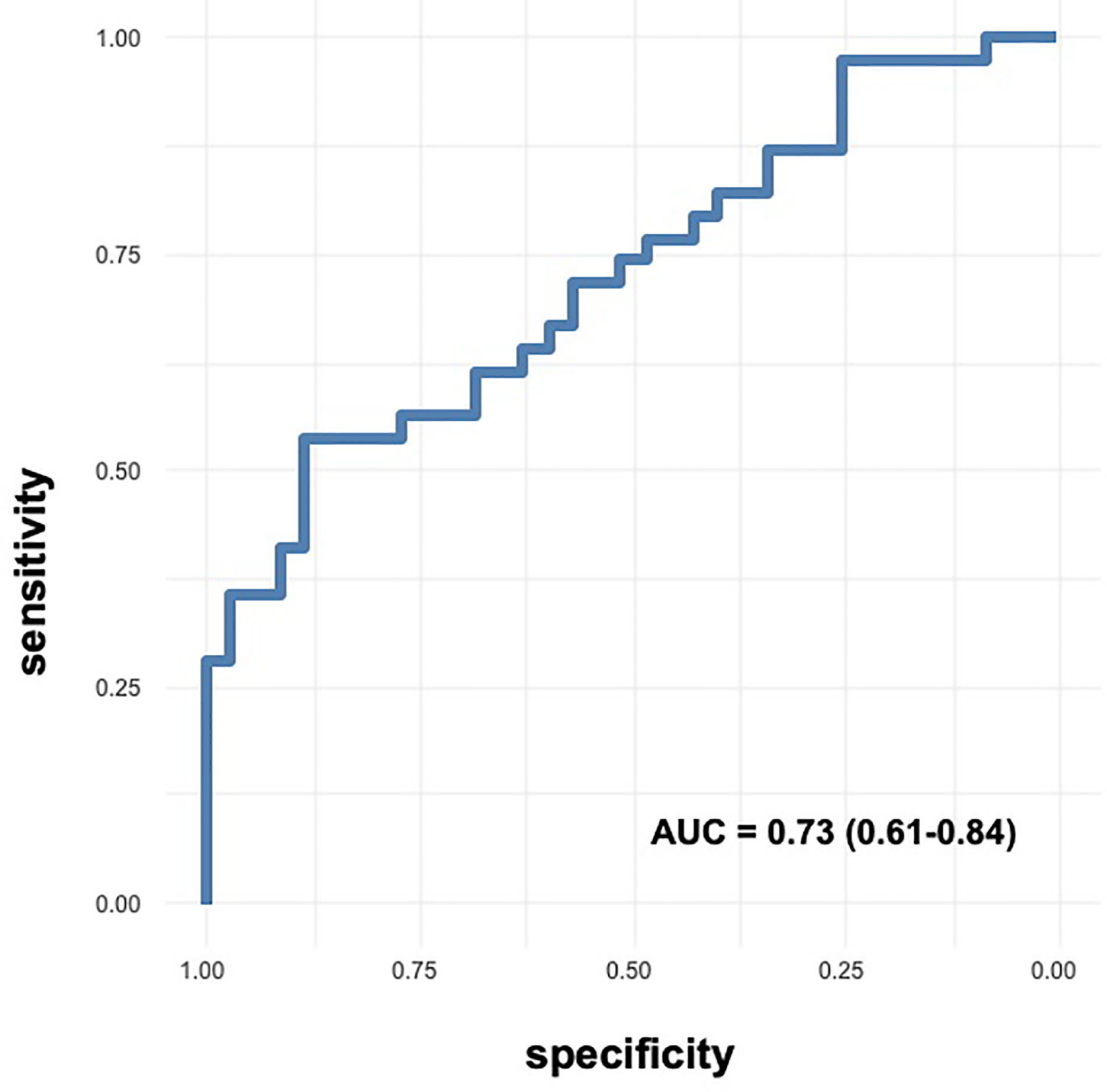
ROC curves of *ACE2* expression in predicting short stature. Accuracy of *ACE2* expression as predictor of short stature.

On ROC curve analysis (**Figure 2**), we found that *ACE2* expression had an AUC of 0.73 (0.61-0.84), indicating a moderate accuracy to predict short stature. We found the same AUC when we considered only the subgroups of 17 controls and 29 cases, as *ACE2* expression had an AUC of 0.73 (0.58-0.88). The cutoff value of *ACE2* expression with highest specificity and sensitivity was 0.73, allowing to correctly classify 63.5% of children with short stature. However, the cutoff value best predicting being a child with short stature was 0.45 fold induction, allowing to classify correctly 70.3% of the children, with sensitivity of 0.54 and specificity of 0.88.

## Discussion

This study shows for the first time that children with short stature, being idiopathic or linked to growth hormone deficiency, had lower *ACE2* expression in PBMC with subsequent increase of the ratio *ACE/ACE2*. *ACE2* expression was associated with the risk of being a children with short stature, regardless of age, sex, and BMI, which suggests that ACE2 should not being related to adiposity, being BMI the most common anthropometric index to estimate adiposity (26). In addition, *ACE2* expression had a moderate accuracy to predict short stature, and the cutoff of 0.45 fold induction was the value of *ACE2* expression best predicting being a child with short stature with specificity of 88% and sensitivity of 54%, allowing to classify correctly 70% of the children.

This finding is consistent with the reports that *ACE2*-knockout mice are smaller than wild-type controls (13–19), and the concept that RAS contributes to growth regulation. Accumulating scientific evidence indicates that RAS is involved in pre-natal growth. Early studies have demonstrated that this system is expressed in both maternal and fetal tissues. AngII levels are higher in uterine venous than arterial blood, or peripheral venous blood in pregnant women (27). The AT1R (AngII type1 receptor) is expressed across all trimesters of pregnancy in the placental syncytiotrophoblast, cytotrophoblast, and the fetal vascular endothelium (28). In addition, on the fetal side, autoradiographic analysis of ^125^I-labeled AngII, showed intense binding in the skin, mesenchymal and connective tissues, and skeletal muscle in the later period of gestation, overlapping with the sites reported for IGF-2 in the rat fetus (4). Also *ACE2* and Ang1-7 are expressed in the rat uterus (29). Ghadhanafar et al. found that both *ACE2* and Ang1-7 expression were reduced in the placenta of dexamethasone-exposed rats and this was associated with low birth weight of the offspring. Consistent with this, Bharadwaj et al. showed that ACE2 deficiency resulted in 3-fold higher AngII content in the placenta, and this was associated with reduced gestational weight gain and significant inhibition of fetal growth, as *ACE2*-knockout pups had significantly lower body weight and length than controls (30). It has been argued that ACE2 deficiency might result in inhibition of fetal growth due to the increase of AngII in the placenta, leading to placental ischemia, consistent with the finding that the chorionic villi of the placenta of pre-eclamptic women displayed increased AngII (31). Recently, a negative correlation was found between birth weight centiles and circulating ACE2 levels (32), depending on ACE2 shedding and tissue loss (33).

Nevertheless, in our study, the children with short stature had no history of being small for gestational age babies, suggesting that they suffered from a post-natal growth defect, which is also influenced by the RAS activity. Animal studies have shown that an infusion of AngII markedly reduced plasma IGF-1 levels (by 56% after 1 week of treatment and by 41% after 2 weeks of treatment) with a parallel reduction of hepatic *IGF-1* mRNA, and body weight, which decreased by 18% after 1 week of treatment (34). These effects were mediated by the AT1R, as they were blocked by its antagonist losartan (34). Consistent with these findings, also the treatment with ACE inhibitors for 3 years was associated with significantly higher levels of IGF-1 in a cohort of 1154 subjects aged > 65 years (35). Nevertheless, in our study, blood pressure as well as circulating AngII and Ang1-7 levels did not significantly differ between the groups. This suggest that the mechanisms underlying the association between ACE2 deficiency and short stature might involve an unbalance on tissue - rather than circulating - AngII and Ang1-7 levels, in organs that are critically involved in growth regulation, such as the pituitary or the liver. For instance, it has been shown that the regulation of somatotrope cell function depends on paracrine processes within the pituitary, which involve peptide hormones such as AngII (36) acting as a signaling molecule (37). In addition to AngII and Ang1-7 tissue levels, ACE2 deficiency affects other peptide hormones, such as atrial natriuretic peptide (ANP). We have shown that both acquired and genetic ACE2 deficiency significantly reduced renal ANP (12), and that tissue ANP production was induced by Ang1-7 (12). ANP reduction could be another mechanism underlying the association between ACE2 reduction and growth retardation, given that natriuretic peptides stimulate endochondral bone growth in animal studies, and natriuretic peptides have gained increasing attention as potential stimulants to skeletal growth (38).

Apart from short stature, *ACE2* expression was influenced by the sex of participants, being higher in the female group, as we have recently found in a cohort of young adults (39). This is due to the fact that *ACE2* gene is located on the X chromosome, and the X chromosome inactivation, which should silence the transcription from one of the two X chromosomes in female mammalian cells, is often incomplete (40). It has been shown that incomplete X chromosome inactivation affects at least 23% of X-chromosomal genes, resulting in sex-biases in gene expression underlying sex-related phenotypic diversity (40).

The limitations of this study include the fact that *ACE* and *ACE2* expression was measured in PBMC, as they represent the most easily accessible tissue in children to perform these analyses, while *ACE* and *ACE2* gene expression in other tissues and activity levels were not assessed. Another issue is the fact that we measured circulating and not tissue peptides, as this would have required the use invasive procedures. Nevertheless, this is the first study evaluating *ACE2* expression in children with short stature, recruited and managed according to current guidelines in a University Hospital pediatric tertiary care center (1).

In conclusion, our study shows for the first time that *ACE2* expression is significantly lower in children with short stature, with potential diagnostic implications, as *ACE2* expression had a moderate accuracy in predicting short stature. In addition, our findings shed light onto potential mechanisms underlying growth retardation, including changes of angiotensins and natriuretic peptides in the organs regulating skeletal growth.

## Data Availability Statement

The raw data supporting the conclusions of this article will be made available by the authors, upon reasonable request.

## Ethics Statement

The studies involving human participants were reviewed and approved by Institutional Review Board of Institute for Maternal and Child Health IRCCS Burlo Garofolo and Regional Ethics Committee (CEUR-2019-Sper-115). Written informed consent to participate in this study was provided by the participants’ legal guardian/next of kin.

## Author Contributions

GT, BF, and SB, study conception and design, data analysis. FT and BT, data collection, data analysis, database organization, figures and tables. GT, FN, and EB, patient recruitment. FG, statistical analysis. EB and BF, important intellectual content. SB wrote the first draft of the manuscript. All authors contributed to manuscript revision, read, and approved the submitted version.

## Funding

This work was supported by the Ministry of Health, Rome - Italy, in collaboration with the Institute for Maternal and Child Health IRCCS Burlo Garofolo, Trieste – Italy (Ricerca Corrente #30/18) to GT, and by a grant from University of Trieste (Assegno di ricerca Dipartimento di Eccellenza 2019) to SB.

## Conflict of Interest

The authors declare that the research was conducted in the absence of any commercial or financial relationships that could be construed as a potential conflict of interest.

## Publisher’s Note

All claims expressed in this article are solely those of the authors and do not necessarily represent those of their affiliated organizations, or those of the publisher, the editors and the reviewers. Any product that may be evaluated in this article, or claim that may be made by its manufacturer, is not guaranteed or endorsed by the publisher.
